# The External Validation of SACrA (Sex, Albumin, Creatinine, and APACHE II) Score for Predicting Nonemergent Renal Replacement Therapy Initiation: A Retrospective Study Based on the Medical Information Mart for Intensive Care - Version IV Database

**DOI:** 10.7759/cureus.86834

**Published:** 2025-06-26

**Authors:** Saki Yamamoto, Ginga Suzuki, Saria Nishioka, Toshimitsu Kobori, Yuka Masuyama, Hibiki Serizawa, Yoshimi Nakamichi, Mitsuru Honda

**Affiliations:** 1 Critical Care Center, Toho University Omori Medical Center, Tokyo, JPN; 2 Emergency Medicine, Toho University Omori Medical Center, Tokyo, JPN

**Keywords:** : acute kidney injury, acute kidney injury(aki)), icu patients, medical intensive care unit (micu), renal replacement therapy (rrt)

## Abstract

Background and objective

The SACrA [sex, albumin, creatinine, and Acute Physiology and Chronic Health Evaluation II (APACHE II)] score was developed to objectively predict nonemergent renal replacement therapy (RRT) initiation. This study aimed to validate the SACrA score externally by using the Medical Information Mart for Intensive Care - Version IV (MIMIC-IV) database.

Methods

We conducted a retrospective cohort study using the MIMIC-IV database. Patients admitted to the ICU with a total hospital stay of ≥7 days were included, whereas those with chronic kidney disease grade 5, end-stage renal disease, post-kidney transplant status, or urgent RRT indications were excluded from this study. The primary outcomes were the discrimination [area under the curve (AUC)] and calibration of the SACrA score to predict nonemergent RRT initiation, defined as blood urea nitrogen (BUN) ≥112 mg/dL or oliguria lasting >72 hours.

Results

Among the 16,360 ICU patients who met the inclusion criteria, 14,226 were analyzed after applying the exclusion criteria. Of them, 658 (4.6%) met the criteria for nonemergent RRT initiation, and 351 patients (2.5%) actually received RRT. The SACrA score showed good discrimination, with an AUC of 0.81 in the primary dataset and similar performance across multiple imputed datasets (AUCs ranging from 0.81 to 0.82). Calibration plots indicated that the SACrA score was well-calibrated, with a slope of 1.002 and an intercept of 0.007. Decision curve analysis indicated the potential clinical utility of this score in guiding nonemergent RRT initiation.

Conclusions

The SACrA score is a reliable tool for predicting nonemergent RRT initiation in critically ill patients. Although it accurately identifies high-risk patients, further randomized controlled trials (RCTs) are required to determine whether targeted interventions based on SACrA scores can improve patient outcomes.

## Introduction

Acute kidney injury (AKI) is a common comorbidity in critically ill patients and is often associated with poor outcomes [1-3]. Renal replacement therapy (RRT) remains the primary supportive therapy for patients with AKI; however, the optimal timing for initiating RRT in these patients remains unclear. A synthesis of previous randomized controlled trials (RCTs) [4-8] has suggested that RRT should be avoided in patients likely to recover spontaneously, whereas initiating RRT during nonemergent phases [blood urea nitrogen (BUN) ≥112 mg/dL or oliguria lasting >72 hours] is reasonable for those who require it. Despite these findings, decisions regarding the initiation or avoidance of RRT still rely heavily on clinical judgment, given the lack of objective tools to guide these decisions [9-12]. To better determine the appropriate criteria for initiating RRT in patients with AKI, an objective tool for predicting nonemergent RRT initiation is required.

The SACrA score, developed by our team [11], is based on four readily available variables: sex, serum albumin (Alb), serum creatinine (Cr), and Acute Physiology and Chronic Health Evaluation (APACHE) II score, and serves as a simple and objective tool to predict nonemergent RRT initiation at admission. Although the SACrA score has demonstrated promising predictive performance, the initial validation was performed using a cohort from a single center, which limits the generalizability of the findings. Therefore, further validation using an independent external cohort is necessary. This study aimed to conduct an external validation of the SACrA score using a cohort completely independent of the original developmental cohort.

## Materials and methods

Study design and setting

This retrospective cohort study was conducted using the Medical Information Mart for Intensive Care - Version IV (MIMIC-IV) database, a publicly available, de-identified electronic health record dataset comprising adult patients admitted to the ICU at the Beth Israel Deaconess Medical Center between 2008 and 2019 [13]. Use of the MIMIC-IV database does not require ethical review board approval.

Objective

This study aimed to externally validate the SACrA score for predicting nonemergent initiation of RRT in critically ill patients.

SACrA score application

The SACrA score was calculated for each patient using the original published formula [11]:

SACrA score = (male: -4; female: 0) - 7 × albumin + 4 × creatinine + APACHE II score

Full details of the score derivation are available in the original publication and summarized in Table 1.

**Table 1:** SACrA score details The SACrA score is calculated as APACHE II score + 4 × Cr – 7 × Alb - (male: 4/female: 0) SACrA: sex, albumin, creatinine, and APACHE; Alb: albumin; Cr: creatinine; APACHE: Acute Physiology and Chronic Health Evaluation

Parameters	Score
Sex (male)	–4
Alb	–7
Cr	4
APACHE Ⅱ score	1

Definition of nonemergent RRT initiation

Nonemergent RRT initiation was defined as the presence of either BUN ≥112 mg/dL or oliguria (<500 mL/day) lasting more than 72 hours. These criteria are based on the delayed RRT strategy validated in the AKIKI-2 trial [8].

Participants

Patients enrolled in MIMIC-IV (version 2.2) were selected if they were admitted to an ICU and had a total hospital stay of ≥7 days, the inclusion criteria being the same as those used when developing the SACrA score [11].

Exclusion Criteria

The exclusion criteria were as follows: chronic kidney disease (CKD) grade 5 or end-stage renal disease (ESRD), post-kidney transplant, urgent RRT indication (pH ≤7.15) [4-8], gastrointestinal bleeding, BUN level 112 mg/dL [8,11], and missing or outlier values for BUN or maximum BUN value during hospitalization (BUNmax). CKD, ESRD, post-kidney transplantation status, and gastrointestinal bleeding were identified using the International Classification of Diseases (ICD)-9 and ICD-10 codes listed in Table 2. Missing and outlier values for BUN and BUNmax were included in the exclusion criteria because they directly affected the determination of nonemergent RRT initiation.

**Table 2:** List of ICD-9 and ICD-10 codes used ICD: International Classification of Diseases, Ninth Revision, Clinical Modification; CKD: chronic kidney disease; ESRD: end-stage renal disease

Codes		Diagnosis
ICD-9	ICD-10	
390–459	l00–l99	Cardiovascular diseases
460–519	J00–J99	Respiratory diseases
520–579	K00–K93	Digestive diseases
240–279	E00–E89	Metabolic disorders
001–139	A00–B99	Infections
800–999	S00–T88	Trauma
		Gastrointestinal bleeding
578.0–578.9	K92.0–K92.2	Hematemesis, blood in stool, hemorrhage
530.7	I85.01–I85.11	Bleeding from the esophagus
	K57.21, K57.41	Diverticular bleeding
531.0–531.4, 532.0–532.4, 533.0–533.4, 534.0–534.4	K25.0–K25.4, K26.0–K26.4, K27.0–K27.4, K28.0–K28.4	Gastrointestinal ulcer bleeding
535.01, 535.11, 535.21, 535.41, 535.51	K29.01, K29.51	Gastritis, duodenitis
569.85		Others
585.5, 585.6	N18.5, N18.6	CKD grade 5 and ESRD
996.81–996.84, V42.0	T86.10–T86.13, Z94.0	Post-kidney transplant
584.5–584.9, 669.32, 997.5	N17.0–N17.9, O90.4, T79.5	Acute kidney injury

Data extraction

The following data were extracted from the medical records: age, sex, BMI, cause of admission (ICD-9 and ICD-10 codes used are listed in Table 2), vital signs, and blood tests, including sodium, potassium, Alb, BUN, BUNmax, Cr, white blood cell count, hematocrit, pH, partial pressure of carbon dioxide, partial pressure of oxygen (PaO_2_), fraction of inspired oxygen (FiO_2_), and bicarbonate levels. Vital signs and blood test results were extracted as the earliest values within the first 24 hours of admission. If data from the emergency department were available, the values were used. The PaO_2 _and FiO_2 _pairs were included if their recording times differed by >2 hours. The APACHE II scores were calculated for each variable and tabulated. When calculating the Cr item, the presence or absence of AKI was determined using ICD-9 and ICD-10 codes (as listed in Table 2). However, points for chronic conditions were not included because accurately determining the presence of chronic pathologies was challenging.

Outcome

The primary outcomes of this study were discrimination and calibration of the SACrA score.

Sample size estimation

Given the large sample size available in the database, it was considered sufficient to validate the predictive score. Therefore, prior sample size calculations were not performed. However, we used the C-statistic [area under the curve (AUC)] and outcome incidence rates determined during the validation process of the predictive score to calculate the required sample size post hoc using the pmsampsize package in R [14].

Outlier and missing values

The MIMIC-IV database contains many outliers and missing values. Outliers were identified based on the clinical criteria, and the definitions and counts of outliers for each variable are listed in Table 3. The outliers were excluded and treated as missing data. Missing data, including outliers treated as missing values, were imputed using the mice package in R. The classification and regression trees method was used to predict missing values based on the observed data [15-16]. The imputation process was conducted over 10 iterations, generating five imputed datasets (Datasets 1-5). The numbers of missing values for each variable are shown in Table 3. Given that variations in the handling of outliers and missing values could potentially impact the analysis results, Datasets 1-5 were used to assess the robustness and validity of the findings.

**Table 3:** Summary of missing and outlier values GCS: Glasgow Coma Scale; SBP: systolic blood pressure; DBP: diastolic blood pressure; Alb: albumin; BUN: blood urea nitrogen; Cr: creatinine; WBC: white blood cell; Ht: hematocrit

Parameters	Missing values	Outlier values	Definition of outlier
Age, n (%)	0 (0%)	0 (0%)	≥92
Sex, n (%)	0 (0%)	Not applicable	Not applicable
Body mass index, n (%)	5,125 (36.0%)	209 (1.5%)	≥50.0, ≤12.0
Vital signs			
GCS, n (%)	17 (0.1%)	0 (0%)	≥16, ≤2
Body temperature, n (%)	85 (0.6%)	315 (2.2%)	≥44.0, ≤20.0
SBP, n (%)	2,231 (15.7%)	284 (2.0%)	≥250, ≤49
DBP, n (%)	2,229 (15.7%)	498 (3.5%)	≥180, ≤29
Heart rate, n (%)	1 (0.01%)	241 (1.7%)	≥250, ≤25
Respiratory rate, n (%)	3 (0.02%)	1701 (12.0%)	≥50, ≤4
Blood tests			
Sodium, n (%)	2 (0.01%)	3 (0.02%)	≥180, ≤89
Potassium, n (%)	22 (0.2%)	1 (0.01%)	≥8.0, ≤0.9
Alb, n (%)	3,285 (23.1%)	5224 (36.7%)	≥6.0, ≤0.5
BUN, n (%)	Excluded	Excluded	≤0.9
BUNmax, n (%)	Excluded	Excluded	99,999, ≤0.9
Cr, n (%)	3 (0.02%)	9 (0.06%)	≥12.0, ≤0.09
WBC, n (%)	97 (0.7%)	11 (0.08%)	≥100.0, ≤0.09
Ht, n (%)	3 (0.02%)	22 (0.2%)	≥60.0, ≤10.9
pH, n (%)	2,544 (17.9%)	14 (0.1%)	≥7.57
PaCO_2_, n (%)	2,831 (19.9%)	4 (0.03%)	≥120.0, ≤9.0
PaO_2_, n (%)	5,106 (35.9%)	100 (0.7%)	≥650.0, ≤29.0
FiO_2_, n (%)	5,106 (35.9%)	80 (0.6%)	≤0.2
HCO_3_, n (%)	22 (0.2%)	0 (0%)	≥46.0, ≤3.0

Statistical analyses

Dataset 1 was used as a representative dataset to provide basic characteristics. Continuous variables are presented as medians and interquartile ranges (IQRs). Categorical variables are presented as percentages. The SACrA score was calculated for all patients in Dataset 1. Receiver operating characteristic (ROC) analysis was performed to assess the discriminatory power of the SACrA score, and the AUC was calculated. The Youden index was used to determine the cutoff values. Additionally, a calibration plot was created to evaluate the model calibration, and metrics such as R², slope, intercept, and Brier score were calculated. To verify the adequacy of the imputations, the discrimination and calibration of the SACrA score were similarly assessed and compared using the imputed datasets (Datasets 2-5). Furthermore, the clinical utility of the SACrA score was evaluated using decision curve analysis (DCA), which visually represents net benefit [17]. Statistical analyses were performed using R version 4.2.0, and statistical significance was set at p <0.05.

## Results

**Figure 1:**
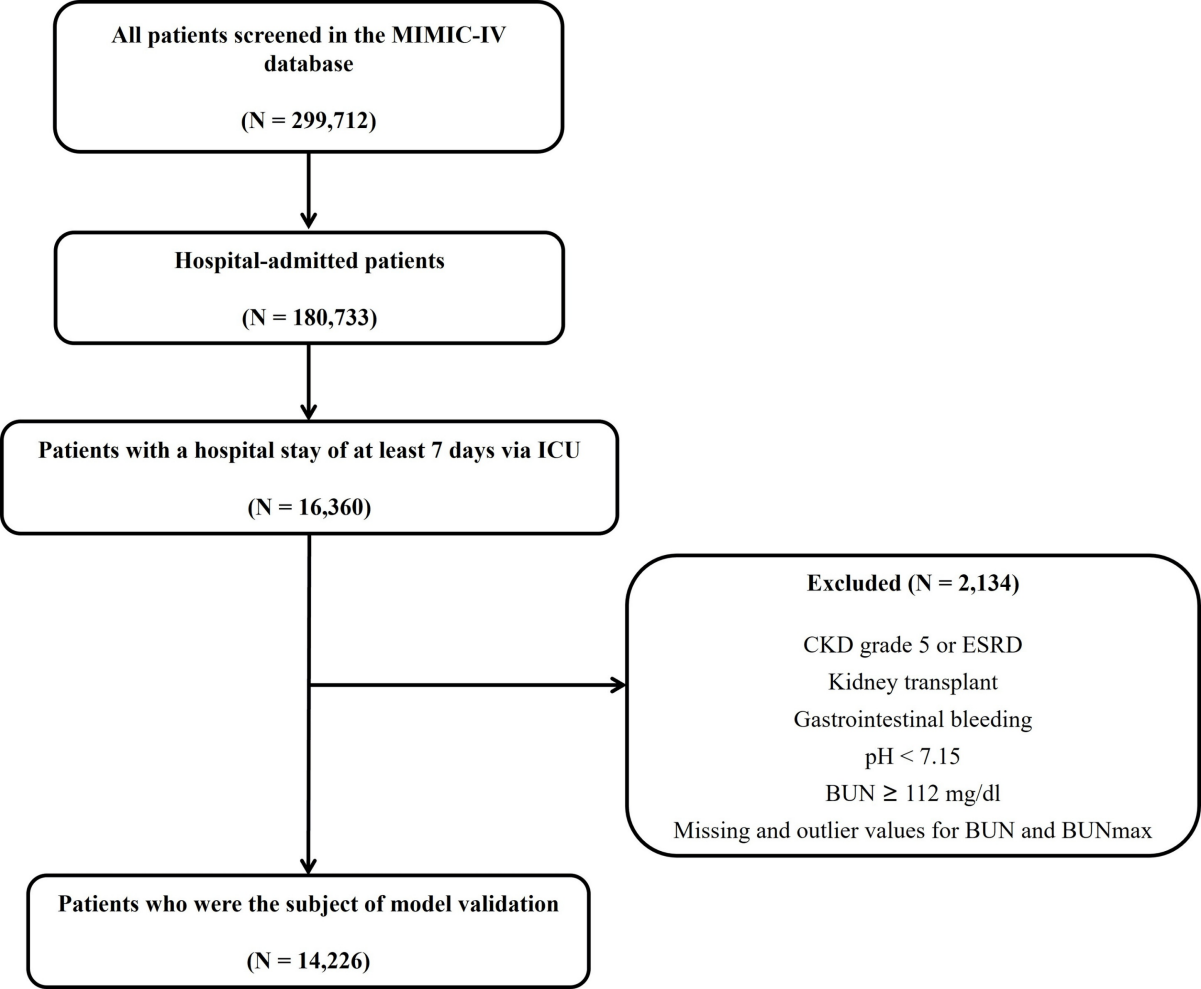
Patient flow diagram MIMIC: Medical Information Mart for Intensive Care; ICU: intensive care unit; CKD: chronic kidney disease; ESRD: end-stage renal disease; BUN: blood urea nitrogen

Among the 299,712 patients included in MIMIC-IV version 2.2, 16,360 were admitted to the ICU with a total hospital stay of ≥7 days. After applying the exclusion criteria, 14,226 patients were included in the analysis (Figure 1). Among the excluded patients, 2,134 were removed due to outlier or missing BUN/BUNmax values.

Baseline characteristics for Dataset 1 (the representative imputed dataset) are shown in Table 4. The median (IQR) age was 66 (54-77) years, and 8,220 (57.8%) were male. The median SACrA score was 2.9 (-5.2-10.6). A total of 658 patients (4.6%) met the criteria for nonemergent RRT initiation, among whom 360 (2.5%) met only the oliguria criterion, 182 (1.3%) only the BUN criterion, and 116 (0.8%) both (Table 5). Of these, 351 patients (2.5%) actually underwent RRT.

**Table 4:** Patient characteristics GCS: Glasgow Coma Scale; SBP: systolic blood pressure; DBP: diastolic blood pressure; Alb: albumin; BUN: blood urea nitrogen; Cr: creatinine; WBC: white blood cell; Ht: hematocrit; APACHE: Acute Physiology and Chronic Health Evaluation

Parameters	Values (N = 14,426)
Age, years, median (IQR)	66.0 (54.0–77.0)
Sex (male), n (%)	8220 (57.8%)
Body mass index, kg/m^2^,median (IQR)	27.5 (23.9–32.0)
Cause of admission, n (%)	
Cardiovascular	4961 (34.9%)
Respiratory	159 (1.1%)
Digestive	85 (0.6%)
Metabolic	105 (0.7%)
Infection	2002 (14.1%)
Trauma	6802 (47.8%)
Others	107 (0.8%)
Unknown	5 (0.04%)
Vital signs, median (IQR)	
GCS	7.0 (3.0–14.0)
Body temperature, ℃	36.3 (35.9–36.5)
SBP, mmHg	95.0 (83.0–112.0)
DBP, mmHg	49.0 (42.0–60.0)
Heart rate, /min	65.0 (57.0–75.0)
Respiratory rate, /min	12.0 (9.0–15.0)
Blood tests, median (IQR)	
Sodium, mEq/l	136.0 (133.0–138.0)
Potassium, mEq/l	3.4 (3.2–3.7)
Alb, mg/dl	3.0 (2.5–3.5)
BUN, mg/dl	14.0 (9.0–20.0)
BUNmax, mg/dl	26.0 (19.0–40.0)
Cr, mg/dl	0.7 (0.6–1.0)
WBC, ×10^3/μl	8.3 (6.2–10.8)
Ht, %	27.5 (23.5–32.3)
pH	7.4 (7.3–7.4)
PaCO_2_, torr	34.0 (30.0–39.0)
PaO_2_, torr	146.0 (80.0–257.0)
FiO_2_	0.6 (0.5–1.0)
HCO_3_, mEq/l	21.0 (19.0–23.0)
APACHE Ⅱ score	23.0 (16.0–29.0)

**Table 5:** Summary of SACrA score and nonemergent RRT initiation criteria SACrA: sex, albumin, creatinine, and Acute Physiology and Chronic Health Evaluation; RRT: renal replacement therapy; BUN: blood urea nitrogen

Parameters	Values (N = 14,426)
SACrA score, median (IQR)	2.9 (–5.2–10.6)
Nonemergent RRT initiation, n (%)	658 (4.6%)
Oliguria lasting > 72 hours	360 (2.5%)
BUNmax ≥112 mg/dL	182 (1.3%)
Both	116 (0.8%)
Actual RRT initiation, n (%)	351 (2.5%)

**Figure 2:**
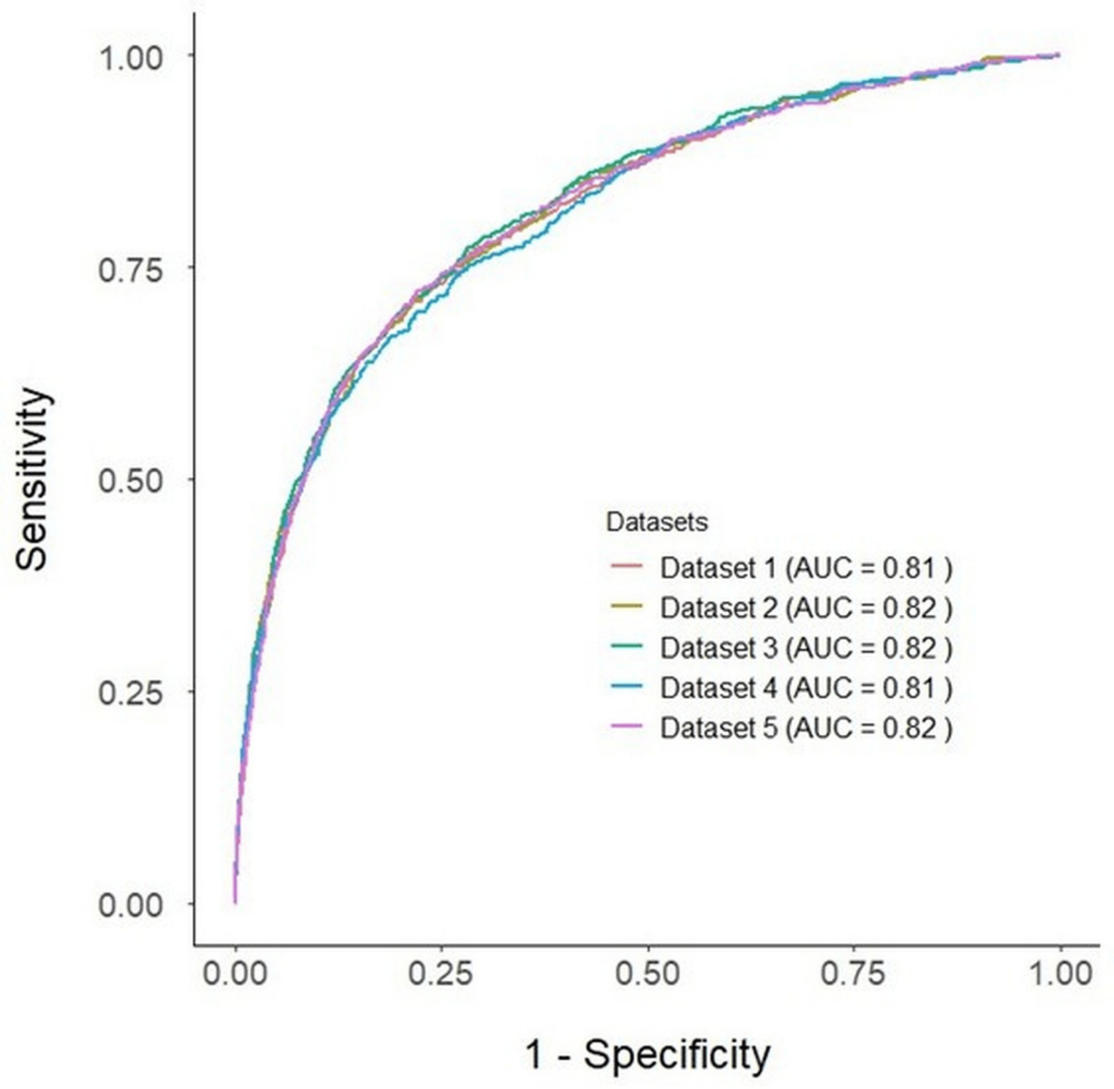
ROC curves of each dataset ROC curves for prediction scores in five datasets generated by multiple imputation. The AUC for Datasets 1 and 4 was 0.81, and for Datasets 2, 3, and 5, it was 0.82 ROC: receiver operating characteristic; AUC: area under the curve

The SACrA score demonstrated good discrimination with an AUC of 0.81 in Dataset 1, and consistent AUC values across Datasets 2-5 (ranging from 0.81 to 0.82) (Figure 2). Cutoff values determined using the Youden index ranged from 11.1 to 12.3 across datasets, indicating stability of the score's predictive threshold.

**Figure 3:**
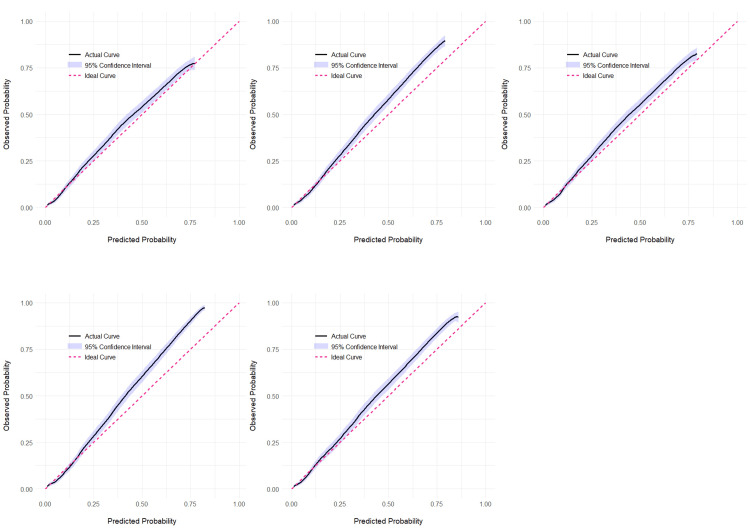
Calibration plot of each dataset Calibration curves for prediction scores in five datasets generated by multiple imputation. (a) The calibration curve for Dataset 1, with an intercept of 0.004 and a slope of 1.000. (b) The calibration curve for Dataset 2, with an intercept of 0.004 and a slope of 1.001. (c) The calibration curve for Dataset 3, with an intercept of 0.002 and a slope of 1.000. (d) The calibration curve for Dataset 4, with an intercept of 0.001 and a slope of 1.000. (e) The calibration curve for Dataset 5, with an intercept of –0.002 and a slope of 0.999

Calibration plots confirmed strong calibration of the SACrA score (Figure 3). In Dataset 1, the calibration slope was 1.002 and the intercept was 0.007, both of which are close to ideal values, indicating good agreement between the predicted and observed risks. R² was 0.214, and the Brier score was 0.039. Calibration performance was similarly acceptable in all datasets (Table 6).

**Table 6:** Performance of SACrA score AUC: area under the curve; SACrA: sex, albumin, creatinine and Acute Physiology and Chronic Health Evaluation

Parameters	Dataset 1	Dataset 2	Dataset 3	Dataset 4	Dataset 5
Discrimination					
AUC	0.81	0.82	0.82	0.81	0.82
Calibration					
Slope	1.002	1.002	1.002	1.001	1.000
Intercept	0.007	0.009	0.008	0.003	0.000
R^2^	0.214	0.223	0.225	0.215	0.217
Brier	0.039	0.038	0.039	0.039	0.039

**Figure 4:**
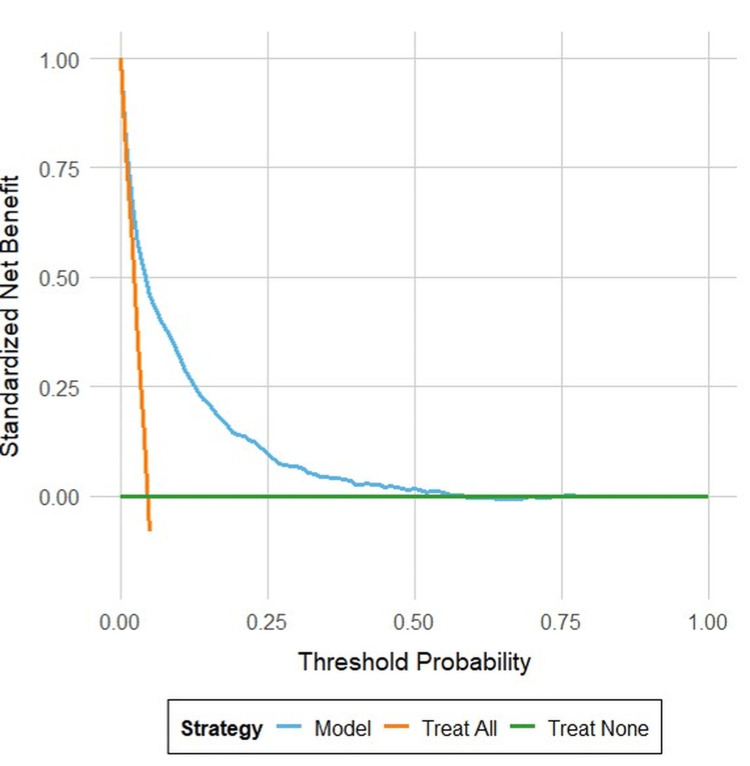
Decision curve analysis for clinical decision-making The figure presents a decision curve analysis comparing three strategies: using the predictive score, treating all patients, and treating no patients. The blue line represents the net benefit of using the predictive score, the orange line represents treating all patients, and the green line represents treating no patients. The analysis shows that using the predictive score is beneficial at all threshold probabilities

Decision curve analysis (DCA) showed that using the SACrA score resulted in higher net benefit than either treating all or no patients across clinically relevant threshold probabilities (Figure 4), supporting its potential clinical utility.

A post hoc sample size calculation based on the AUC (0.81), number of predictors (four), and event rate (4.6%) suggested that a minimum of 559 patients would be required, confirming that our sample size was sufficient for external validation.

## Discussion

This study aimed to validate the external validity of the SACrA score in predicting nonemergent RRT initiation using the MIMIC-IV database. The SACrA score demonstrated good discrimination and calibration among the patients in the MIMIC-IV database, indicating its potential clinical utility. The SACrA score may aid in avoiding unnecessary RRT, potentially contributing to improved patient outcomes.

Previous research

Several studies have proposed models to predict the need for RRT in patients with AKI; however, many of these models have limitations in their predictive performance or lack external validation [18-20]. The SACrA score has been internally validated using bootstrapping methods, and its good performance has been confirmed through external validation in an independent cohort. Additionally, the SACrA score was developed using a cohort that strictly adhered to the nonemergent RRT initiation criteria following a delayed strategy, making it particularly suitable for predicting nonemergent RRT. In this study, further external validation was conducted using an entirely different database. These findings suggest that the SACrA score may apply to various clinical settings.

Strengths and clinical impact

Firstly, this study utilized the MIMIC-IV database and a sufficiently large sample size to robustly evaluate the generalizability of the SACrA score. Previously, the performance of the SACrA score in populations entirely different from that of the development cohort was unclear [11]. However, this study demonstrates that the SACrA score is applicable in diverse clinical settings, including facilities with patients of various racial backgrounds. Second, the SACrA score is straightforward to calculate using data collected in the emergency department. Calculation of the SACrA score can include the APACHE II score based on the initial data obtained before ICU admission. This enables the early prediction of nonemergent RRT initiation during hospitalization. By identifying patients who met the criteria for nonemergent RRT initiation, the SACrA score serves as a tool to evaluate the effectiveness of early interventions in these patients. Third, outliers were managed from a clinical perspective, and missing values were imputed using multiple imputations. Five imputed datasets were generated, and the performance of the SACrA score was assessed across each dataset, demonstrating robust results. Fourth, the clinical utility of the SACrA score was confirmed using the DCA. Using this score to avoid unnecessary RRT can benefit patients and potentially improve their outcomes.

Interpretation

As previously reported, Alb and Cr levels reflect inflammation, nutritional status, and renal reserve [21-22]. The formula for the SACrA score suggests that a low Alb level is strongly associated with worsening of kidney function, whereas Cr serves as a direct indicator of renal function. The APACHE II score represents the overall severity of illness, particularly reflecting the risk of multiple organ failure [23]. Patients with higher disease severity are at an increased risk of organ failure, including AKI, which likely increases the need for RRT. The SACrA score indicates that AKI is closely related to the overall condition of the patient and serves as a tool to better understand the interplay between AKI and systemic health status.

Limitations

This study has several limitations. First, although data were extracted from a well-established database, a substantial number of outliers and missing values were present. We addressed this issue by excluding implausible values based on clinical judgment and using multiple imputation to handle missing data, which allowed for robust analysis. In addition, because we included only patients with a total hospital stay of ≥7 days, selection bias may have occurred, potentially excluding patients with mild AKI who recovered early or those with rapid deterioration and early mortality. Second, diagnoses were identified using ICD codes, which may not fully capture disease severity or all relevant conditions. For instance, chronic health components of the APACHE II score could not be reliably included, potentially affecting model performance. Third, the SACrA score tended to underestimate risk in high-risk patients. This may reflect model overfitting in the development cohort. Future strategies such as recalibration using larger multi-center cohorts or the inclusion of dynamic clinical variables may help address this issue. Finally, although this external validation was conducted using a large U.S.-based cohort, further validation is warranted in other populations, including those in Japan, to confirm broader generalizability. Additionally, randomized controlled trials are needed to determine whether clinical decision-making based on the SACrA score improves patient outcomes.

## Conclusions

The SACrA score demonstrated good performance in predicting nonemergent RRT initiation in a large, independent ICU cohort. While the findings support its potential clinical utility, further prospective validation and RCTs are required to confirm its impact on patient outcomes and applicability across different populations.
